# Homologs of the yeast Tvp38 vesicle-associated protein are conserved in chloroplasts and cyanobacteria

**DOI:** 10.3389/fpls.2013.00467

**Published:** 2013-11-18

**Authors:** Rebecca Keller, Dirk Schneider

**Affiliations:** Department of Pharmacy and Biochemistry, Johannes Gutenberg-UniversityMainz, Germany

**Keywords:** biogenesis, DedA, membrane structure, thylakoid membrane, Tvp38, vesicle transfer

## Abstract

Vesicle transfer processes in eukaryotes depend on specific proteins, which mediate the selective packing of cargo molecules for subsequent release out of the cells after vesicle fusion to the plasma membrane. The protein Tvp38 is conserved in yeasts and higher eukaryotes and potentially involved in vesicle transfer processes at the Golgi membrane. Members of the so-called “SNARE-associated proteins of the Tvp38-family” have also been identified in prokaryotes and those belong to the DedA protein family. Tvp38/DedA proteins are also conserved in cyanobacteria and chloroplasts. While only a single member of this family appears to be present in chloroplasts, cyanobacterial genomes typically encode multiple homologous proteins. Mainly based on our understanding of the DedA-homologous proteins of *Escherichia coli*, it appears likely that the function of these proteins in chloroplast and cyanobacteria involves stabilizing and organizing the structure of internal membrane systems.

## Tvp38—a vesicle-associated protein of the golgi compartment

Vesicle transfer processes are involved in diverse transport events in eukaryotic cells, such as uptake of compounds into a cell or protein secretion. Many factors, involved in vesicle formation and budding as well as in membrane fusion, have been identified and characterized in the last decades and details of vesicle transport mechanisms are understood on the molecular level (Bonifacino and Glick, 2004; Foresti and Denecke, 2008). In the secretory pathway, proteins are co-translationally synthesized into the lumen of the endoplasmic reticulum (ER) and subsequently transported in various vesicle transfer steps from the ER via the Golgi apparatus to the plasma membrane. Vesicle transfer along the secretory pathway depends on specific proteins, which mediate the selective packing of cargo molecules for subsequent release out of the cells after vesicle fusion to the plasma membrane. While some proteins are directly involved in membrane fusion, other proteins are crucial for vesicle formation, cargo selection, vesicle budding or for selective intracellular targeting and transport of vesicles (Rothman and Wieland, 1996; Bonifacino and Glick, 2004).

**Figure 1:**
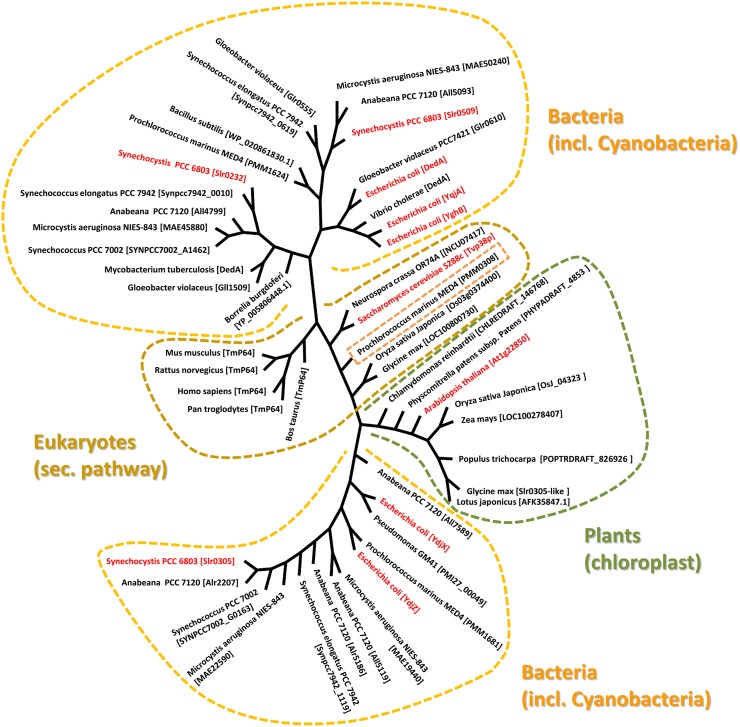
**Evolutionary relationship between Tvp38-homologs.** The phylogenetic tree was generated using selected Tvp38/DedA-homologs from different species using the Cobalt Constraint-based Multiple Protein Alignment Tool (http://www.ncbi.nlm.nih.gov/tools/cobalt/cobalt.cgi). To visualize relationships among the various DedA-homologous proteins, an unrooted tree with unscaled branches is shown. A rooted tree with scaled branches is given in the supplementary figure S1.

In a proteomic analysis of a *Saccharomyces cerevisiae* Golgi subcompartment membrane fraction, which was defined by the vesicle-fusion protein Tlg2 (Abeliovich et al., 1998), several membrane-associated proteins have been identified, including the transmembrane protein Tvp38 (Tlg2-compartment vesicle protein of 38 kDa) (Inadome et al., 2005). While Tvp38 is not essential for growth of the yeast *S. cerevisiae* under laboratory conditions (Inadome et al., 2007), its co-localization with other proteins involved in vesicular membrane trafficking suggests an important function in membrane transport. In line with this, homologs of Tvp38 are not only conserved in fungi but also in higher eukaryotes, including humans (Inadome et al., 2007) (Figure 1). Although the exact physiological role of Tvp38 remains elusive, a putative role in cargo selection was implicated (Inadome et al., 2007).

## Vesicle transfer in chloroplasts and cyanobacteria

Intra-plastidial vesicular transfer processes have been discussed for a long time, although the molecular mechanisms and proteins potentially involved are mainly uncharacterized. Early electron microscopy analyses already indicated a *de novo* formation of thylakoid membranes, and the thylakoid membrane network is built up by fusion of vesicular structures budding from the chloroplast inner envelope membrane (von Wettstein, 2001; Vothknecht and Westhoff, 2001). However, such a mechanism involves controlled and aligned processes, including formation of inner envelope vesicles, distinct packing of proteins and lipids as well as controlled vesicle fusion. While similar steps are discussed in case of the secretory pathway (Bonifacino and Glick, 2004), essentially nothing is known about the molecular details in chloroplasts. Formation of vesicular structures at the inner envelope membrane has been observed under defined experimental conditions (Westphal et al., 2001a), and a protein of about 30 kDa has been discussed to be involved in this process. Consequently, the protein has been named vesicle-inducing protein in plastids 1 (Vipp1) (Kroll et al., 2001). In fact, depletion of this protein results in disturbed thylakoid membrane formation in *Arabidopsis thaliana* (Kroll et al., 2001). A Vipp1-homolog is also conserved in cyanobacteria, which share a common ancestor with chloroplasts, and depletion of Vipp1 results in disturbed thylakoid membrane assembly also in cyanobacteria (Westphal et al., 2001b; Fuhrmann et al., 2009; Gao and Xu, 2009). However, thus far vesicular structures have not been described in cyanobacteria, and it appears likely that, in contrast to chloroplasts, thylakoid membranes are not built *de novo* in cyanobacteria (Barthel et al., 2013). Thus, the physiological function of Vipp1 might be different than vesicle formation (Bultema et al., 2010; Vothknecht et al., 2012). Furthermore, beside Vipp1, no proteins (eventually) involved in membrane trafficking within chloroplasts or cyanobacteria have been experimentally identified so far. However, bioinformatic analyses identified several proteins in the predicted *A. thaliana* proteome with homology to factors involved in vesicle transport along the secretory pathway (Andersson and Sandelius, 2004; Khan et al., 2013). Among others, a SNARE-associated protein with homology to the Tvp38 has been identified, which contains an N-terminal chloroplast targeting sequence, and thus the protein is very likely localized within chloroplasts (Khan et al., 2013).

## Tvp38-homologs in chloroplasts and cyanobacteria

**Figure 2:**
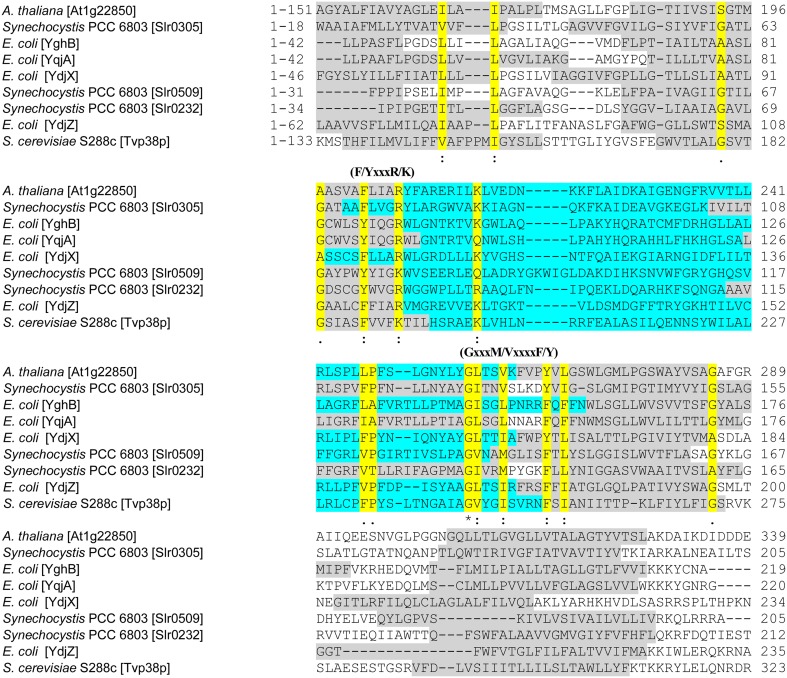
**Comparative alignment for chloroplast and cyanobacterial Tvp38/DedA-homologs.** In the multi-sequence alignment, selected parts of the amino acid sequences of Slr0305, Slr0509, Slr0232 and the chloroplast SNARE-associated protein At1g22850 of *Arabidopsis thaliana* (Khan et al., 2013) are shown. Other amino acid sequences used in the alignment are the Tvp38-protein of *Saccharomyces cerevisiae* (S288c) (Inadome et al., 2007) and the best characterized prokaryotic DedA-homologs YghB, YqjA, YdjX and YdjZ of *E. coli* (Doerrler et al., 2013). The multi-sequence alignment was performed using CLUSTAL 2.1 (Larkin et al., 2007; Goujon et al., 2010). Prediction of transmembrane segments (shown in gray) were conducted using TMHMM (Sonnhammer et al., 1998). Conserved regions are highlighted in yellow and marked with one dot = conserved; two dots = highly conserved or with an asterisk = identical. The predicted, conserved loop regions are highlighted in turquoise. Regions, which are, based on HMM-Logo prediction, conserved in the Tvp38/DedA family (PF09335) (Punta et al., 2012), are marked above the corresponding sequences as (F/YxxxR/K) and (GxxxM/VxxxxF/Y).

The so-called SNARE-associated proteins of the Tvp38-family constitute a largely uncharacterized protein family. Results from bioinformatic analyses indicate that a protein with similarities to Tvp38 is conserved in plant chloroplasts (Khan et al., 2013) (Figure 1), and indeed the protein from *A. thaliana* has already been identified in an analysis of the *A. thaliana* chloroplasts proteome (Zybailov et al., 2008). In contrast to chloroplasts, which harbor only a single Tvp38-homolog, multiple Tvp38-homologs are typically encoded in cyanobacterial genomes (Figure 1, Table S1). Cyanobacteria share a common ancestor with chloroplasts of higher plants or algae, and they represent popular and accessible model systems to investigate physiological processes associated with thylakoid membranes. In the genome of the thus far best characterized cyanobacterium *Synechocystis* sp. PCC 6803 (hereafter *Synechocystis*), three genes encode SNARE-associated Tvp38-homologs (Kaneko et al., 1996), which are classified as members of the DedA protein family in bacteria (Liang et al., 2010). The *Synechocystis* genes *slr0232*, *slr0305* and *slr0509*, which are all localized in distinct and separate gene loci, encode membrane integral proteins with polypeptide lengths of 218, 209 and 205 amino acids (aa's), respectively. For both, Slr0232 and Slr0305, five transmembrane helices are predicted by the program TMHMM (Sonnhammer et al., 1998), whereas Slr0509 has only four predicted transmembrane helices (Figure 2). However, a conserved domain of a canonical LeuT-fold is predicted by computational methods for the bacterial Tvp38-homologous proteins of the DedA protein family (Khafizov et al., 2010). Two repeats of a domain of five transmembrane helices together form the full LeuT-fold found in many functional transport proteins, such as the bacterial homolog of sodium-dependent neurotransmitter transporters (Yamashita et al., 2005). Thus, it appears to be likely that also Slr0509 contains five transmembrane segments, not all of them being predicted by computational methods.

Thus far, only Slr0232 has been identified in a proteomic analysis of *Synechocystis* membrane integral proteins, and an important function of this protein in stress-adaptive processes is indicated (Qiao et al., 2013). Parts of a loop region in Slr0232 (aa's 79–110) exhibit 37% sequence identity to a GTPase activator protein from *A. thaliana* (AT5G52580.1), which is involved in assembly of the vesicle coat in the eukaryotic secretory system. As the *Synechocystis* genome encodes multiple small G-proteins with largely uncharacterized functions (Kaneko et al., 1996), Slr0232 might be involved in membrane organization together with a small G-protein. The *Synechocystis* protein Slr0305 shows high similarity to a predicted protein of *A. thaliana* (At5g19070.1), which is annotated as a SNARE-associated Golgi protein. Thus, this further links Slr0305 to membrane transport processes.

Slr0509 and Slr0232 are closely related proteins and share 29% sequence identity. Therefore, these two proteins cluster together in the phylogenetic tree (Figure 1). In contrast, Slr0305 appears to be closer related to the Tvp38-homologs found in chloroplasts (Figure 1). The single DedA-homologous protein of *Borrelia burgdorferi*, which is essential for cell viability (Liang et al., 2010; Doerrler et al., 2013), clusters together with the *Synechocystis* proteins Slr0232 and Slr0509. These findings implicate an essential function of Tvp38/DedA-homologous proteins within this branch. In fact, at least one Slr0232/Slr0509-homolog appears to be conserved in cyanobacteria (Table S1), except in case of the not well characterized *Synechococcus* strains JA-2-3Bá(2–13) and JA-3-3Ab. As individual cyanobacterial strains contain only a single protein with homology to Slr0232 or Slr0509 it appears likely that the proteins Slr0232 and Slr0509 have overlapping and redundant functions, whereas members of the Slr0305-defined cyanobacterial branch might exhibit distinct physiological functions. Multiple Slr0305-homologous proteins with potentially overlapping functions were identified in several cyanobacteria (Table S1), whereas only in case of the atypical cyanobacterium *Gloeobacter violaceus* sp. PCC 7421, which completely lacks an internal thylakoid membrane system, no Slr0305-homologous protein has been predicted (Rippka et al., 1974). Noteworthy, the cyanobacterial Tvp38/DedA homologs are located in two different clusters in a phylogenetic tree (Figure 1), in line with earlier observations (Boughner and Doerrler, 2012). The *Synechocystis* proteins Slr0305 and Slr0232/Slr0509, respectively, are representatives of these two clusters. Slr0232 and Slr0509 cluster in close proximity to the *Escherichia coli* YghB and YqjA proteins, which are the so far best characterized proteins of the prokaryotic DedA protein family (Doerrler et al., 2013). In contrast, Slr0305 of *Synechocystis* clusters together with the *E. coli* YdjX and YdjZ proteins. While these latter *E. coli* proteins are both clearly annotated as members of the Tvp38 family, they might fulfill different functions than the YqjA and YghB proteins of *E. coli*. In fact, complementation studies, using the temperature-sensitive *E. coli* mutant strain BC202 (see below), have shown that YdjX and YdjZ were not capable of restoring an observed cell division defect, in contrast to other putative DedA-homologous proteins of *E. coli* (Boughner and Doerrler, 2012).

Members of the Tvp38/DedA protein family have a characteristic region, which has been annotated as the DedA domain in case of bacterial DedA-homologous proteins (Doerrler et al., 2013). This region, which is preserved in all three *Synechocystis* homologs as well as in the chloroplast SNARE-associated Golgi protein Atlg22850 of *A. thaliana*, contains parts of the predicted transmembrane segments (Figure 2, gray) and the encompassed loop region (Figure 2, turquoise). A highly conserved glycine in a predicted amphipathic region has been previously identified as a prominent feature of the bacterial DedA family of Tvp38-homologous proteins (Doerrler et al., 2013), and this glycine is also conserved in the chloroplast and in cyanobacterial homologs. However, this glycine appears to be part of a larger, conserved sequence motif GxxxM/VxxxxF/Y (Figure 2), and in the Tvp38-homologs of eukaryotes, bacteria, chloroplasts and cyanobacteria a F/YxxxR/K motif appears to be conserved as well (Figure 2). The exact function of the identified motifs is elusive. Besides being of direct functional importance, the conserved amino acids could be involved in folding and stabilization of individual proteins or higher oligomeric states, as dimerization of Tvp38/DedA proteins has been discussed previously (Khafizov et al., 2010). However, the function of the cyanobacterial and chloroplast Tvp38/DedA-homologs is completely elusive, whereas members of this family have been analyzed to some extent in other bacteria.

## Members of the bacterial DedA protein family of Tvp38-homologous proteins are involved in membrane organization

The bacterial and archaeal members of Tvp38-homologous proteins are annotated as members of the DedA protein family (Doerrler et al., 2013). The number of putative DedA-homologs varies significantly between different species (Khafizov et al., 2010; Liang et al., 2010; Doerrler et al., 2013). Deletion of the single *dedA* gene *bb0250* in *Borrelia burgdorferi* resulted in cell death (Liang et al., 2010), and combined deletion of the eight genes encoding putative DedA-homologs in *E. coli* revealed a lethal growth defect (Boughner and Doerrler, 2012). Thus, while multiple DedA-homologs might have redundant and overlapping functions, the presence of at least one functional DedA protein appears to be essential. This is an interesting observation, as previous studies indicated that the eukaryotic DedA-homolog Tvp38 is non-essential in yeast (Inadome et al., 2007). Thus, evolution of more complex membrane targeting mechanisms in eukaryotes may have resulted in a dispensable function of the DedA/Tvp38 proteins.

The function of prokaryotic DedA-homologs is clearly linked to processes at the cytoplasmic membrane and/or biogenesis of the whole cell envelope (Shi et al., 2004; Ledgham et al., 2005; Sikdar and Doerrler, 2010; Sikdar et al., 2013). The *E. coli* DedA-homologs YqjA and YghB are the best investigated members of the DedA protein family so far (Thompkins et al., 2008; Liang et al., 2010; Sikdar and Doerrler, 2010; Sikdar et al., 2013). The temperature-sensitive *E. coli* strain BC202, in which the two genes *yqjA* and *yghB* are deleted, displays a cell division defect, which is caused by the mislocalization of two periplasmic amidases (Thompkins et al., 2008; Sikdar and Doerrler, 2010). Importantly, these amidases are required for remodeling of the peptidoglycan layer during growth (Ize et al., 2003) and were not efficiently transported into the periplasm of the mutant strain BC202 via the Twin arginine transport (Tat) pathway (Sikdar and Doerrler, 2010). The Tat-pathway specifically transports folded proteins across the cytoplasmic membrane of bacteria and archaea as well as across the chloroplast thylakoid membrane (Palmer and Berks, 2012). Furthermore, BC202 displays an altered membrane lipid composition with decreased levels of the zwitterionic phospholipid phosphatidylethanolamine (PE) and increased levels of the acidic phospholipids phosphatidylglycerol (PG) and cardiolipin (Thompkins et al., 2008; Liang et al., 2010). This indicates an important role of the two DedA-homologs as to the membrane architecture. Earlier studies, using *E. coli* mutant strains defective in synthesis of PE and anionic phospholipids, respectively, revealed that lipid polymorphism is needed for an effective protein transport via the Tat-pathway, and thus the altered lipid composition in *E. coli* BC202 will affect Tat-dependent transport processes (Mikhaleva et al., 1999). Moreover, alterations of the proton motive force (*pmf*) were also observed in *E. coli* BC202 (Sikdar et al., 2013), and both, the altered phospholipid content and the disturbed *pmf*, might influence the function and dynamics of the Tat-pathway. As there is no indication of YqjA and/or YghB being directly involved in phospholipid synthesis, deletion of the two encoding genes has most likely destabilized the membrane, resulting in a decreased *pmf* and the need for decreasing the level of the non-bilayer forming (membrane destabilizing) phospholipid PE and increasing the level of the bilayer-forming phospholipid PG. In line with the assumption that the membrane integrity and/or organization is disturbed, the *psp* (phage shock protein) response pathway is also activated in BC202 (Sikdar et al., 2013), which is well known to control the expression of genes in response to diverse membrane stress-inducing conditions (Darwin, 2005). Furthermore, the Cpx-system was also highly activated in BC202 (Sikdar et al., 2013), which also controls transcription of genes controlling membrane integrity (Hunke et al., 2012; Raivio et al., 2013). In fact, transcription of *yqjA* in *E. coli* is regulated by the Cpx-signal transduction pathway (Yamamoto and Ishihama, 2006; Price and Raivio, 2009). Together, all these observations support the assumption that the *E. coli* DedA proteins YqjA and YghB are required for membrane stability, integrity and/or organization.

## Putative functions of Tvp38/DedA proteins in chloroplasts and cyanobacteria

The functions of Tvp38 in yeast and mammals as well as of the homologous DedA proteins of bacteria and archaea are still not well understood. The initial studies in yeast have indicated that Tvp38 plays a role in vesicular trafficking along the secretory pathway and might be involved in organizing vesicular structures (Inadome et al., 2005, 2007). Most studies of the *E. coli* DedA proteins have linked the proteins' functions to the plasma membrane (Doerrler et al., 2013). While some analyses could support the idea that DedA proteins might be directly involved in transport processes across the bacterial membrane (Ledgham et al., 2005; Barabote et al., 2006; Sikdar et al., 2013), it currently appears most likely that DedA proteins are involved in stabilization and/or organization of the prokaryotic cytoplasmic membrane, and indirectly affect other membrane-associated processes, such as solute uptake or protein transport via the Tat-pathway (Sikdar and Doerrler, 2010). Together, the function of all Tvp38/DedA proteins is clearly linked to membrane-associated processes, and thus we propose that also in chloroplasts and cyanobacteria the homologous proteins are involved in maintaining the integrity of the internal membranes.

Involvement of the cyanobacterial and chloroplast proteins in lipid and/or protein exchange between the inner envelope/cytoplasmic membrane and the thylakoid membrane appears likely, based on the early observations that Tvp38 is involved in formation of vesicles in the late Golgi compartment. Interestingly, the Tvp38/DedA-homolog PMM0308 of *Prochlorococcus marinus* MED4 clusters together with the eukaryotic Tvp38 proteins of the secretory pathway (Figure 1), suggesting a physiological function similar to Tvp38. Moreover, while all analyzed cyanobacterial species contained at least one protein homologous to the *Synechocystis* Slr0305 protein, solely in the genome of *Gloeobacter violaceus* PCC 7421 no homologous protein has been identified (Table S1). *Gloeobacter* is the only cyanobacterium identified thus far, which does not have an internal thylakoid membrane system (Rippka et al., 1974). This observation might link the function of the Slr0305-homologous proteins to thylakoid membranes. Either Slr0305-like proteins might be directly involved in maintaining the stability and/or architecture of thylakoid membranes or the proteins are involved in transport processes from the inner envelope membrane in chloroplasts or the cyanobacterial cytoplasmic membrane to internal thylakoid membranes, respectively. Noteworthy, besides the Tvp38-homologs, other proteins with homology to proteins involved in exocytic vesicular trafficking have been identified to reside within chloroplasts (Andersson and Sandelius, 2004; Khan et al., 2013). Thus, a series of proteins, including the here discussed Tvp38-homolog, might play a role in initiation, assembly, budding/tethering of vesicles and/or membrane fusion. Nevertheless, further experiments are required to clearly classify the plant and cyanobacterial Tvp38/DedA proteins, especially to understand their role in membrane integrity, membrane transport and/or fusion. Analyzing the sub-cellular membrane location of the Tvp38/DedA-homologs as well as putative redundant and overlapping functions of the multiple cyanobacterial proteins will be a first step to understand their precise role in the biogenesis and/or integrity of thylakoid membranes.

## Supplementary material

The Supplementary Material for this article can be found online at: http://www.frontiersin.org/journal/10.3389/fpls.2013.00467/abstract

## Conflict of interest statement

The authors declare that the research was conducted in the absence of any commercial or financial relationships that could be construed as a potential conflict of interest.

## References

[B1] Abeliovich H. Grote E. Novick P. Ferro-Novick S. (1998). Tlg2p, a yeast syntaxin homolog that resides on the Golgi and endocytic structures. J. Biol. Chem. 273, 11719–11727 10.1074/jbc.273.19.11719 9565594

[B2] Andersson M. X. Sandelius A. S. (2004). A chloroplast-localized vesicular transport system: a bio-informatics approach. BMC Genomics 5:40 10.1186/1471-2164-5-40 15236667 PMC481061

[B3] Barabote R. D. Tamang D. G. Abeywardena S. N. Fallah N. S. Fu J. Y. Lio J. K. (2006). Extra domains in secondary transport carriers and channel proteins. Biochim. Biophys. Acta. 1758, 1557–1579 10.1016/j.bbamem.2006.06.018 16905115

[B4] Barthel S. Bernat G. Seidel T. Rupprecht E. Kahmann U. Schneider D. (2013). Thylakoid membrane maturation and PS II activation are linked in greening Synechocystis sp. PCC (6803). cells. Plant Physiol. 163, 1037–1046 10.1104/pp.113.224428 23922268 PMC3793023

[B5] Bonifacino J. S. Glick B. S. (2004). The mechanisms of vesicle budding and fusion. Cell 116, 153–166 10.1016/S0092-8674(03)01079-1 14744428

[B6] Boughner L. A. Doerrler W. T. (2012). Multiple deletions reveal the essentiality of the DedA membrane protein family in *Escherichia coli*. Microbiology 158, 1162–1171 10.1099/mic.0.056325-0 22301910

[B7] Bultema J. B. Fuhrmann E. Boekema E. J. Schneider D. (2010). Vipp1 and PspA: related but not twins. Commun. Integr. Biol. 3, 162–165 10.4161/cib.3.2.10529 20585511 PMC2889975

[B8] Darwin A. J. (2005). The phage-shock-protein response. Mol. Microbiol. 57, 621–628 10.1111/j.1365-2958.2005.04694.x 16045608

[B9] Doerrler W. T. Sikdar R. Kumar S. Boughner L. A. (2013). New functions for the ancient DedA membrane protein family. J. Bacteriol. 195, 3–11 10.1128/JB.01006-12 23086209 PMC3536176

[B10] Foresti O. Denecke J. (2008). Intermediate organelles of the plant secretory pathway: identity and function. Traffic 9, 1599–1612 10.1111/j.1600-0854.2008.00791.x 18627574

[B11] Fuhrmann E. Gathmann S. Rupprecht E. Golecki J. Schneider D. (2009). Thylakoid membrane reduction affects the photosystem stoichiometry in the *cyanobacterium Synechocystis sp*. PCC (6803). Plant Physiol. 149, 735–744 10.1104/pp.108.132373 19109411 PMC2633836

[B12] Gao H. Xu X. (2009). Depletion of Vipp1 in Synechocystis sp. PCC (6803). affects photosynthetic activity before the loss of thylakoid membranes. FEMS Microbiol. Lett. 292, 63–70 10.1111/j.1574-6968.2008.01470.x 19222583

[B13] Goujon M. McWilliam H. Li W. Valentin F. Squizzato S. Paern J. (2010). A new bioinformatics analysis tools framework at EMBL-EBI. Nucleic Acids Res. 38(Suppl.)W695–W699 10.1093/nar/gkq313 20439314 PMC2896090

[B14] Hunke S. Keller R. Müller V. S. (2012). Signal integration by the Cpx-envelope stress system. FEMS Microbiol. Lett. 326, 12–22 10.1111/j.1574-6968.2011.02436.x 22092888

[B15] Inadome H. Noda Y. Adachi H. Yoda K. (2005). Immunoisolation of the yeast Golgi subcompartments and characterization of a novel membrane protein, Svp26, discovered in the Sed5-containing compartments. Mol. Cell. Biol. 25, 7696–7710 10.1128/MCB.25.17.7696-7710.2005 16107716 PMC1190314

[B16] Inadome H. Noda Y. Kamimura Y. Adachi H. Yoda K. (2007). Tvp38, Tvp23, Tvp18 and Tvp15: novel membrane proteins in the Tlg2-containing Golgi/endosome compartments of *Saccharomyces cerevisiae*. Exp. Cell Res. 313, 688–697 10.1016/j.yexcr.2006.11.008 17178117

[B17] Ize B. Stanley N. R. Buchanan G. Palmer T. (2003). Role of the *Escherichia coli* Tat pathway in outer membrane integrity. Mol. Microbiol. 48, 1183–1193 10.1046/j.1365-2958.2003.03504.x 12787348

[B18] Kaneko T. Sato S. Kotani H. Tanaka A. Asamizu E. Nakamura Y. (1996). Sequence analysis of the genome of the unicellular *cyanobacterium Synechocystis* sp. strain PCC6803. II. Sequence determination of the entire genome and assignment of potential protein-coding regions. DNA Res. 3, 185–209 10.1093/dnares/3.3.185 8905238

[B19] Khan N. Z. Lindquist E. Aronsson H. (2013). New putative chloroplast vesicle transport components and cargo proteins revealed using a bioinformatics approach: an arabidopsis Model. PLoS ONE 8:e59898 10.1371/journal.pone.0059898 23573218 PMC3613420

[B20] Khafizov K. Staritzbichler R. Stamm M. Forrest L. R. (2010). A study of the evolution of inverted-topology repeats from LeuT-fold transporters using AlignMe. Biochemistry 49, 10702–10713 10.1021/bi101256x 21073167

[B21] Kroll D. Meierhoff K. Bechtold N. Kinoshita M. Westphal S. Vothknecht U. C. (2001). VIPP1, a nuclear gene of *Arabidopsis thaliana* essential for thylakoid membrane formation. Proc. Natl. Acad. Sci. U.S.A. 98, 4238–4242 10.1073/pnas.061500998 11274447 PMC31209

[B22] Larkin M. A. Blackshields G. Brown N. P. Chenna R. McGettigan P. A. McWilliam H. (2007). ClustalW and ClustalX version 2. Bioinformatics 23, 2947–2948 10.1093/bioinformatics/btm404 17846036

[B23] Ledgham F. Quest B. Vallaeys T. Mergeay M. Covès J. (2005). A probable link between the DedA protein and resistance to selenite. Res. Microbiol. 156, 367–374 10.1016/j.resmic.2004.11.003 15808941

[B24] Liang F. T. Xu Q. Sikdar R. Xiao Y. Cox J. S. Doerrler W. T. (2010). BB0250 of Borrelia burgdorferi is a conserved and essential inner membrane protein required for cell division. J. Bacteriol. 192, 6105–6115 10.1128/JB.00571-10 20870761 PMC2981195

[B25] Mikhaleva N. I. Santini C. L. Giordano G. Nesmeyanova M. A. Wu L. F. (1999). Requirement for phospholipids of the translocation of the trimethylamine N-oxide reductase through the Tat pathway in *Escherichia coli*. FEBS Lett. 463, 331–335 10.1016/S0014-5793(99)01661-0 10606748

[B26] Palmer T. Berks B. C. (2012). The twin-arginine translocation (Tat) protein export pathway. Nat. Rev. Microbiol. 10, 483–496 10.1038/nrmicro2814 22683878

[B27] Price N. L. Raivio T. L. (2009). Characterization of the Cpx regulon in *Escherichia coli* strain MC4100. J. Bacteriol. 191, 1798–1815 10.1128/JB.00798-08 19103922 PMC2648356

[B28] Punta M. Coggill P. C. Eberhardt R. Y. Mistry J. Tate J. Boursnell C. (2012). The Pfam protein families database: Nucleic Acids Res. 40, D290–D301 10.1093/nar/gkr1065 22127870 PMC3245129

[B29] Qiao J. Shao M. Chen L. Wang J. Wu G. Tian X. (2013). Systematic characterization of hypothetical proteins in Synechocystis sp. PCC (6803). reveals proteins functionally relevant to stress responses. Gene 512, 6–15 10.1016/j.gene.2012.10.004 23063937

[B30] Raivio T. L. Leblanc S. K. Price N. L. (2013). The *Escherichia coli* Cpx envelope stress response regulates genes of diverse function that impact antibiotic resistance and membrane integrity. J. Bacteriol. 195, 2755–2767 10.1128/JB.00105-13 23564175 PMC3697260

[B31] Rippka R. Waterbury J. Cohen-Bazire G. (1974). A cyanobacterium which lacks thylakoids. Arch. Microbiol. 100, 419–436 10.1007/BF00446333

[B32] Rothman J. E. Wieland F. T. (1996). Protein sorting by transport vesicles. Science 272, 227–234 10.1126/science.272.5259.227 8602507

[B33] Sikdar R. Doerrler W. T. (2010). Inefficient Tat-dependent export of periplasmic amidases in an *Escherichia coli* strain with mutations in two DedA family genes. J. Bacteriol. 192, 807–818 10.1128/JB.00716-09 19880597 PMC2812453

[B34] Sikdar R. Simmons A. R. Doerrler W. T. (2013). Multiple envelope stress response pathways are activated in an *Escherichia coli* strain with mutations in two members of the DedA membrane protein family. J. Bacteriol. 195, 12–24 10.1128/JB.00762-12 23042993 PMC3536178

[B35] Shi Y. Cromie M. J. Hsu F. F. Turk J. Groisman E. A. (2004). PhoP-regulated Salmonella resistance to the antimicrobial peptides magainin 2 and polymyxin B. Mol. Microbiol. 53, 229–241 10.1111/j.1365-2958.2004.04107.x 15225317

[B36] Sonnhammer E. L. von Heijne G. Krogh A. (1998). A hidden Markov model for predicting transmembrane helices in protein sequences. Proc. Int. Conf. Intell. Syst. Mol. Biol. 6, 175–182 9783223

[B37] Thompkins K. Chattopadhyay B. Xiao Y. Henk M. C. Doerrler W. T. (2008). Temperature sensitivity and cell division defects in an *Escherichia coli* strain with mutations in yghB and yqjA, encoding related and conserved inner membrane proteins. J. Bacteriol. 190, 4489–4500 10.1128/JB.00414-08 18456815 PMC2446817

[B38] Yamamoto K. Ishihama A. (2006). Characterization of copper-inducible promoters regulated by CpxA/CpxR in *Escherichia coli*. Biosci. Biotechnol. Biochem. 70, 1688–1695 10.1271/bbb.60024 16861804

[B39] Yamashita A. Singh S. K. Kawate T. Jin Y. Gouaux E. (2005). Crystal structure of a bacterial homologue of Na+/Cl–dependent neurotransmitter transporters. Nature 437, 215–223 10.1038/nature03978 16041361

[B40] Vothknecht U. C. Westhoff P. (2001). Biogenesis and origin of thylakoid membranes. Biochim. Biophys. Acta 1541, 91–101 10.1016/S0167-4889(01)00153-7 11750665

[B41] Vothknecht U. C. Otters S. Hennig R. Schneider D. (2012). Vipp1: a very important protein in plastids?!. J. Exp. Bot. 63, 1699–1712 10.1093/jxb/err357 22131161

[B42] von Wettstein D. (2001). Discovery of a protein required for photosynthetic membrane assembly. Proc. Natl. Acad. Sci. U.S.A. 98, 3633–3635 10.1073/pnas.071056598 11274378 PMC33329

[B43] Westphal S. Soll J. Vothknecht U. C. (2001a). A vesicle transport system inside chloroplasts. FEBS Lett. 506, 257–261 10.1016/S0014-5793(01)02931-3 11602257

[B44] Westphal S. Heins L. Soll J. Vothknecht U. C. (2001b). Vipp1 deletion mutant of Synechocystis: a connection between bacterial phage shock and thylakoid biogenesis. Proc. Natl. Acad. Sci. U.S.A. 98, 4243–4248 10.1073/pnas.061501198 11274448 PMC31210

[B45] Zybailov B. Rutschow H. Friso G. Rudella A. Emanuelsson O. Sun Q. (2008). Sorting signals, N-terminal modifications and abundance of the chloroplast proteome. PLoS ONE 3:e1994 10.1371/journal.pone.0001994 18431481 PMC2291561

