# Supplementary material for: Homologs of the yeast Tvp38 vesicle-associated protein are conserved in chloroplasts and cyanobacteria
Source: Front Plant Sci. 2013 Nov 18;4:467. doi: 10.3389/fpls.2013.00467 (PMC3836016; doi:10.3389/fpls.2013.00467)
Supplement: Supplementary file 1 [file DataSheet1.PDF]

Table S1 Cyanobacterial Tvp38/DedA-homologs.

Orthologs search identified by using (Pfam PF09335) and found by BLAST using the database Cyanobase (Nakao *et al.*, 2010).

The identities [in %] of orthologous proteins in relation to Slr0305, Slr0232 or Slr0509 of *Synechocystis* PCC 6803 are indicated. No Tvp38/DedA-homologs were identified for *Thermosynechococcus elongatus* BP-1.

Cyanobacterium	Orthologs	% Identity	Orthologs	% Identity	Orthologs	% Identity
**Synechocystis PCC 6803**	**Slr0305**		**Slr0232**		**Slr0509**	
**Anabaena PCC 7120**	alr2207	78.6	All4799	53.9	all5093	50.5
	alr5186	58.6				
	all5119	37.8				
	all7589	33.1				
**Gloeobacter violaceus PCC 7421**	---		gll1509	41.2	glr0555	48.5
	---		glr0610	32.4		
**Microcystis aeruginosa NIES-843**	MAE22590	77.8	MAE45880	60.8	MAE50240	60.8
	MAE19440	34				
**Prochlorococcus marinus SS120**	Pro1843	38.1			Pro1785	45.4
**Prochlorococcus marinus MED4**	PMM1681	38.6			PMM1624	45.3
	PMM0308	29.1				
**Prochlorococcus marinus MIT9313**	PMT2186	47.1			PMT0140	47.8
	PMT0305	28.2				

**Synechococcus WH8102**	SYNW2392	45.1			SYNW0120	50.5
**Synechococcus elongatus PCC 6301**	syc0430_d	57.1	syc1487_d	61.2	syc0905_c	53.3
**Synechococcus CC9311**	sync_2809	42.3			sync_0109	49
	sync_0876	24.2				
**Synechococcus PCC 7002**	SYNPCC7002_G0 163	80.4	SYNPCC7002_A 1462	52.1		
**Acaryochloris marina MBIC11017**	AM1_5085	72.2	AM1_4933	48.6		
	AM1_5086	38.9				
	AM1_3651	31.6				
	AM1_3513	35.2				
	AM1_4639	28.8				
	AM1_5179	28 with Ava2345				
**Prochlorococcus marinus NATL2A**	PMN2A 1280	37.5			PMN2A_1201	52
	PMN2A_1711	22.1				
**Anabaena variabilis ATCC 29413**	Ava_2752	76.5	Ava_2069	55.7	Ava_2364	49
	Ava_2775	59.5				
	Ava_2345	37.1				
**Synechococcus CC9902**	Syncc9902 _2208	45.3			Syncc9902_0147	53.7
	Syncc9902_1878	27.7				
**Synechococcus CC9605**	Syncc9605_2554	45.3			Syncc9605_0103	46.8

**Prochlorococcus marinus MIT 9312**	PMT9312_1774	37			PMT9312_1717	43.9
	PMT9312_0310	29.1				
**Synechococcus elongatus PCC 7942**	Synpcc7942_1119	57.1	Synpcc7942 0010	61.2	Synpcc7942_0619	53.3
**Synechococcus JA-2-3B'a(2-13)**	CYB_0557	60.1	---		---	
	CYB_2273	24.3				
**Synechococcus JA-3-3Ab**	CYA 2613	24.3	---		---	
**Prochlorococcus marinus AS9601**	A9601 18911	36.7			A9601_18341	43.6
	A9601_03321	28.2				
**Prochlorococcus marinus MIT 9515**	P9515 18721	38.6			P9515_18131	42.3
	P9515_04161	22				
	P9515_03421	29.5				
**Prochlorococcus marinus MIT 9303**	P9303 29081	46.5			P9303_01771	46
	P9303_20551	24.6				
	P9303_20181	28.2				
Prochlorococcus marinus **NATL1A**	NATL1 21521	38.1			NATL1_20761	50.3
	NATL1_04261	26.3				
	NATL1_03931	28.2				
**Prochlorococcus marinus MIT 9301**	P9301 18721	36.2			P9301_18161	44.7
	P9301_03331	29.1				
Synechococcus **RCC307**	SynRCC307_2414	43.4			SynRCC307_0107	49.2

**Synechococcus WH 7803**	SynWH7803_2431	46.9			SynWH7803_0170	46.5
	SynWH7803_0603	28				
**Prochlorococcus marinus MIT 9215**	P9215 19541	39.1			P9215_18981	43.2
	P9215_03331	28.2				
	P9215_4121	22				
**Prochlorococcus marinus MIT 9211**	P9211 18091	41.1			P9211_17501	52.6
	P9211_03691	27.7				
**Cyanothece ATCC 51142**	cce_4297	57.6	cce_1755	63	cce_3254	46.5
	cce_3995	36.2				
	cce_2348	37.8				
	cce_3820	26.4				
**Nostoc punctiforme ATCC 29133**	Npun_R3471	77.3	Npun_F5562	52.2	Npun_R4473	53
	Npun_R3472	58	Npun_F4627	28.8		
**Cyanothece PCC 7424**	PCC7424_2229	62.8	PCC7424_0186	60.8	PCC7424_1977	63.5
	PCC7424_2230	36.4				
	PCC7424_0620	34.6				
**Cyanothece PCC 7425**	Cyan7425 1944	38.5	Cyan74254907	54.2	Cyan7425_2737	57
	Cyan7425_2175	40.3				

**Cyanothece PCC 8801**	PCC8801_4073 (ok)	59.2	PCC8801_1260	65.6
	PCC8801_4094	37.1		
	PC8801_0962			
**Arthrospira platensis NIES-39**	NIES39_O01540	68.1	NIES39_A00840	61.4
**Trichodesmium erythraeum IMS101**	Tery_1993	66.8		

Figure S1 Phylogenetic tree of selected pro- and eukaryotic DedA-like proteins. The tree is midpoint rooted. The tree is based on a constrain-based multiple sequence alignment (Papadopoulos *et al.*, 2007). For further details see figure 2 and the text.

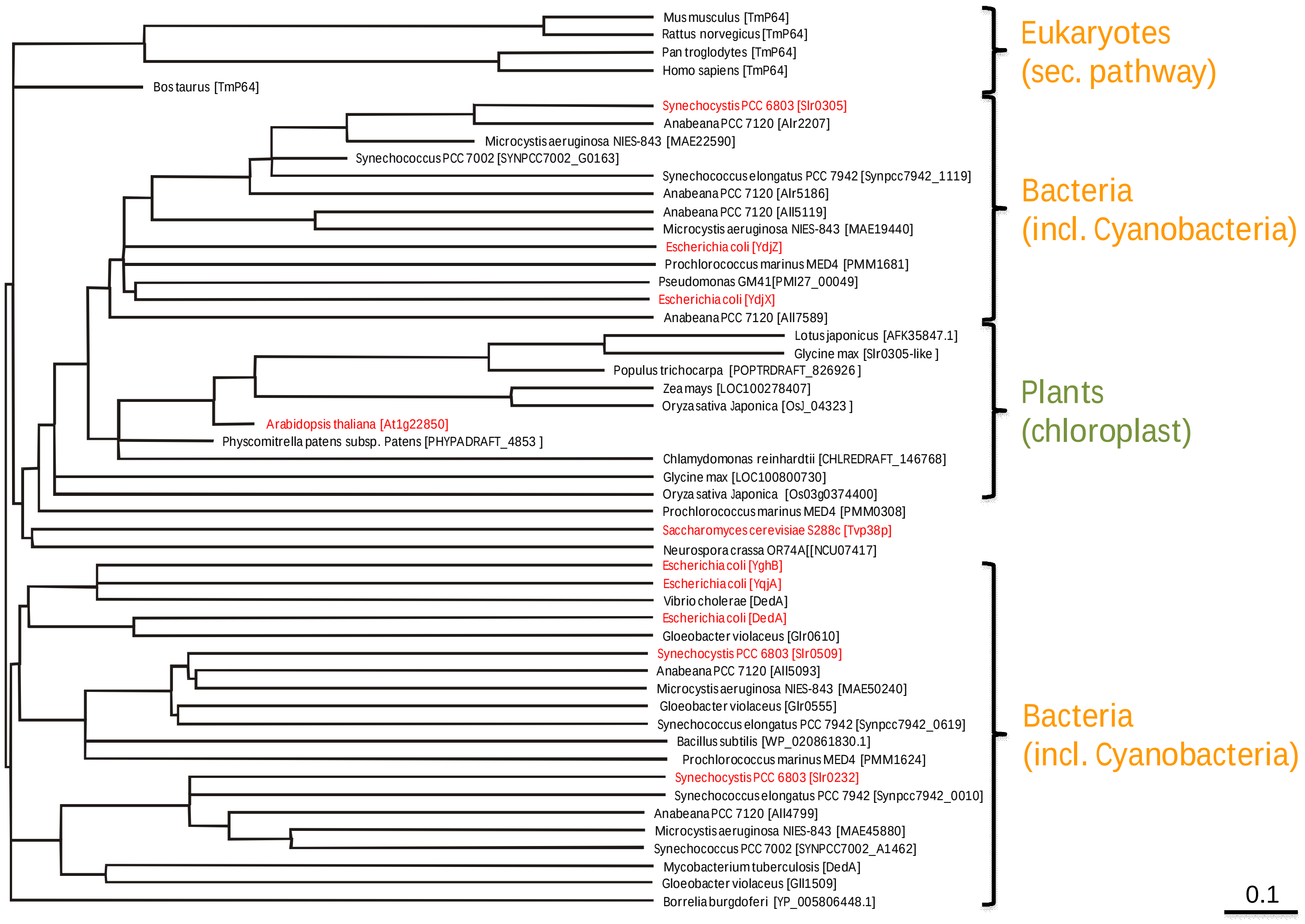

Reference

Nakao. M., Okamoto. S., Kohara. M., Fujishiro. T., Fujisawa. T., Sato. S., Tabata. S., Kaneko. T., and Nakamura. Y. (2010). CyanoBase: the cyanobacteria genome database update 2010. *Nucleic Acids Res.* 38 (Database issue). D379-381.

Papadopoulos. J. S., and Agarwala R. (2007) COBALT: constraint-based alignment tool for multiple protein sequences. *Bioinformatics* 23.1073-1079.
